# Unusual presentation of primary ovarian diffuse large B-cell lymphoma: a case report

**DOI:** 10.1186/s13048-022-00978-2

**Published:** 2022-04-27

**Authors:** Bin Luo, Rong-quan He, Zhi-gang Peng, Jie Ma, Zhen-bo Feng, Gang Chen, Jing-jing Zeng

**Affiliations:** 1 Department of Medical Oncology, First Affiliated Hospital of Guangxi Medical University, No. 6 Shuangyong Road, Nanning, Guangxi Zhuang Autonomous Region 530021 PR China; 2 Department of Pathology, First Affiliated Hospital of Guangxi Medical University, No. 6 Shuangyong Road, Nanning, Guangxi Zhuang Autonomous Region 530021 PR China

**Keywords:** Primary ovarian lymphoma, Diffuse large B-cell lymphoma, Bowel habits

## Abstract

**Background:**

Primary ovarian lymphoma has been difficult to diagnose clinically and pathologically due to its rare incidence and non-specific clinical symptoms.

**Case presentation:**

A 75-year-old female patient was reported in this study. The patient had a six-month history of changes in bowel habits, with occasional black feces and paroxysmal pain in the abdomen. The computed tomography scan of the pelvic cavity illustrated that rectal cancer and sigmoid colon adenocarcinoma invaded the lower part of the right-side ureter. The patient was once treated with excision of part of small intestine, fallopian tube and ovary, and uterus. The pathological examination of these excised tissues, combined with the immunohistochemistry, confirmed that the female patient suffered from primary ovarian diffuse large B-cell lymphoma (DLBCL), and the lymphoma had invaded the entire right-side ovary tissues, serous membranes on the posterior surface of the uterus, and the wall of small intestine.

**Conclusion:**

Few reports were available regarding the primary ovarian DLBCL. The initial symptom of the patient was the changes in bowel habits, which had not been reported beforehand. Hopefully, this case could helpfully render the early diagnosis possible, and increase clinical understanding of primary ovarian DLBCL, which would thereby reduce the chance of misdiagnosis.

## Background

Although diffuse large B-cell lymphoma (DLBCL) has been classified as the most common non-Hodgkin’s lymphoma (NHL) in adults, researchers have rarely diagnosed primary or secondary lymphoma of the ovary, which may lead to misdiagnosis [1]. This study reviewed one case of primary ovarian DLBCL, and the analysis of its clinicopathological features could help to improve our clinical understanding of primary ovarian DLBCL.

## Case presentation

**Fig. 1:**
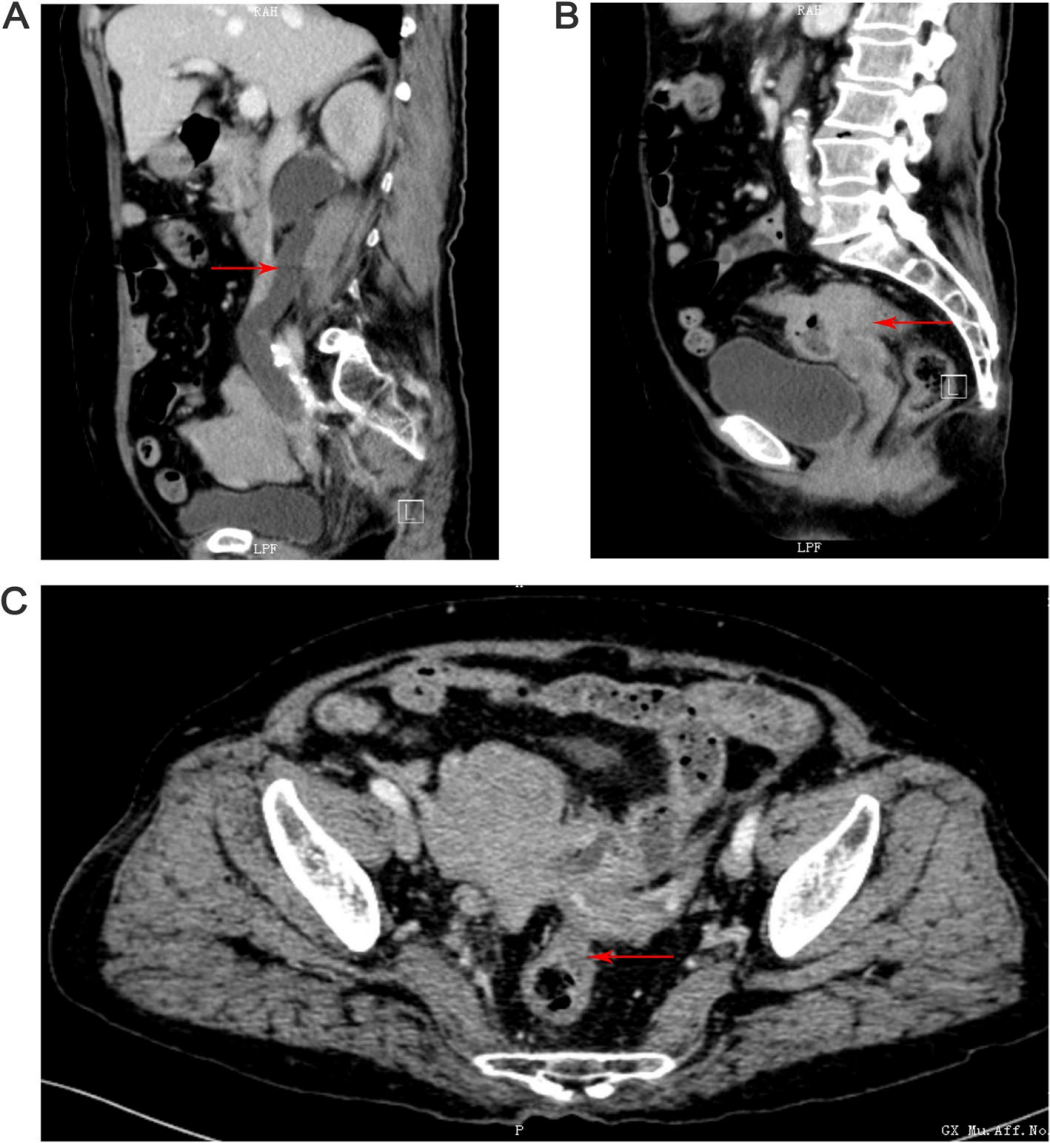
The computed tomography scan of pelvic cavity. **A** The ureterectasis due to compression of large masses in the lower part of the right-side ureter. **B** The upper rectal mass and uterine adhesion were not clearly demarcated. **C** The thickening of the rectal wall and the adhesion of the adnexa are not clearly demarcated. (see red arrows)

A 75-year-old Chinese woman presented with a six-month change in bowel habits for unknown reasons. The symptoms included that the feces appeared thin and dry, and occasionally the feces seemed black, with abdominal pain. However, the patient reported no diarrhea, mucous bloody stool, chills, fever, chest distress, palpitation, or tenesmus. Properly treated at the local hospital, the patient felt somewhat relieved, but she had not received other examination. And we researchers failed to know the details of the treatment. In the First Hospital Affiliated of Guangxi Medical University, colonoscopy showed that rectal mucosal prolapse was examined with characteristics awaiting to be identified, which was pathologically considered as moderate chronic inflammation in rectal mucosa with polypoid hyperplasia, but with no cancer cells detected. The patient was admitted to the hospital for rectal mass. Since the onset of the disease, the patient had low spirits, sleep deprivation, loss of appetite, abnormal feces as mentioned above, weight loss (5 kg less than before), but no abnormalities were observed in the urine. The computed tomography (CT)-scan of pelvic cavity revealed that rectal cancer, together with sigmoid colon adenocarcinoma, invaded the lower part of the right-side ureter, and ureterectasis was also observed on the right (Fig. 1).

The patient was further tests after hospitalization. The physical examination showed T: 37.7℃, P: 94 beats/min, R: 20/min, BP: 169/79 mmHg and without superficial lymphadenopathy. The laboratory test results showed hemoglobin 91.30 g/L, albumin 32.7 g/L and creatinine 95 μmol/L. Besides, her blood electrolyte values, tumor marker routine test, vaginal secretion routine test, coagulation function test, fasting plasma glucose test, urine routine test and stool routine test were within normal limits. Chest X-ray showed no abnormality. After the examination, the exploratory laparotomy was performed with the patient’s consent. The laparotomy disclosed that the right ovary enlarged by the size of 5.0 cm × 3.0 cm, And adhered to the right fallopian tube and the pelvic wall. The tumor was seen to invade the ileum that was approximately 30.0 cm away from the ileocecal region, the intestine seromuscular layer in the mid-rectum, the right-side mesentery, and the site that was 10.0 cm below the lower ureter on the same side. The whole pelvic wall thickened and hardened. The uterus contracted, and no obvious abnormalities were seen in the appearance of the left adnexa. Also laparotomy showed no observable nodules in the liver, spleen, kidneys, mesenteric root of small intestine, greater omentum, or abdominal wall. In the operation, the patient underwent the excision of part of the small intestine, adnexa on both sides, and the uterus. The removed tissues were then pathologically examined–fixed by 10% neutral buffered formalin, embedded by paraffin, serially sectioned into 4 μm thick, and treated with hematoxylin–eosin (H&E) and immunohistochemical (IHC) staining.

**Fig. 2:**
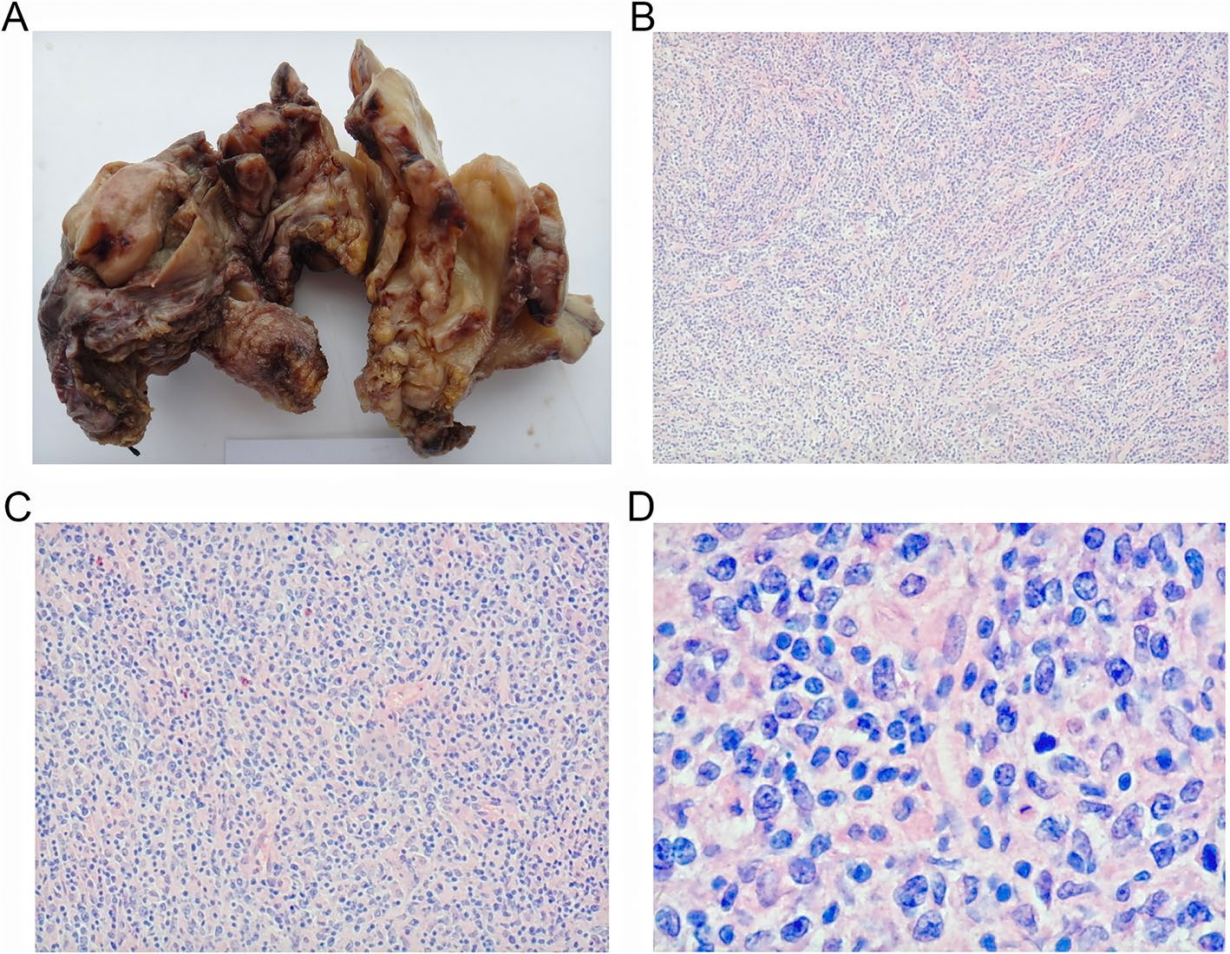
Images of excised tissues of uterus and the both-side adnexa tissues. **A** Macroscopic image of uterus and the both-side adnexa tissues. **B** The microscope revealed the diffusion and infiltration of the tumor cells (H&E × 40). **C** The microscope revealed the diffusion and infiltration of the tumor cells (H&E × 100). **D** The nucleus appeared circular shape, with nuclear division easily visible (H&E × 400). H&E, hematoxylin–eosin

**Fig. 3:**
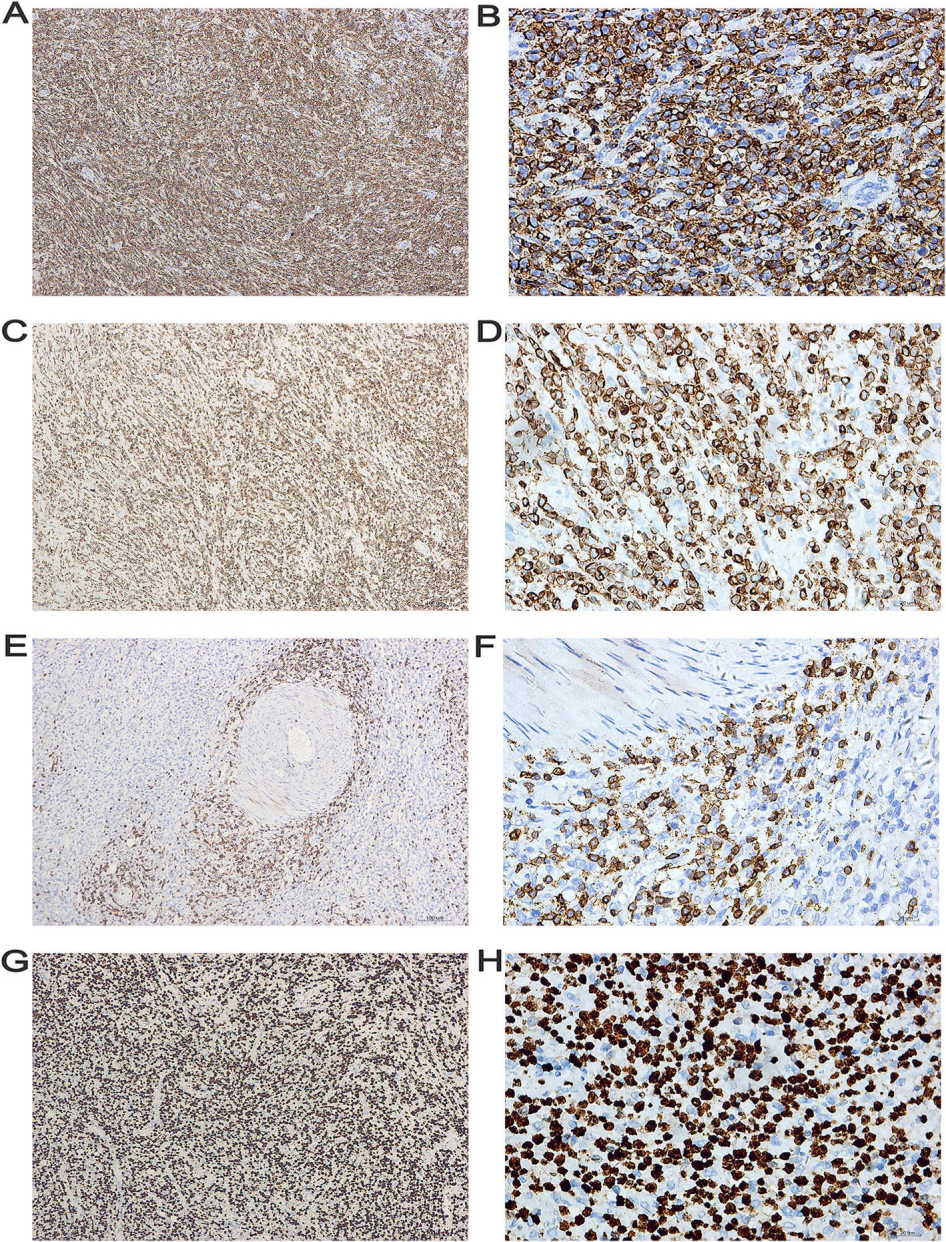
The immunohistochemical characteristics of the ovary tissues under the microscope. In tumor tissues, cytomembrane of CD20 and CD79a were positively expressed: **A** CD20 (DAB × 100). **B** CD20 (× 400). **C** CD79a (DAB × 100). **D** CD79a (× 400). CD3 was negatively expressed: **E** CD3 (DAB × 100). **F** CD3 (× 400). Ki-67 demonstrates aproliferative index of approximately 80%: **G** Ki-67 (DAB × 100). **H** Ki-67 (× 400). DAB, Diaminobenzidine

The gross examination of the uterus and the adnexa: Frozen section procedure was carried out for the right ovary in the operation. The right ovary enlarged, with the size measuring 5.0 cm × 3.0 cm × 3.0 cm. The ovary surface appeared smooth, and the section seemed grey/white, solid, and the texture was moderately soft. No abnormalities were detected in the right adnexa. The uterus measured 7.0 cm × 6.0 cm × 3.0 cm. The perimetrium looked smooth, and two grey-white nodules (0.60 cm – 1.0 cm in diameter) were observed in the serous membranes on the posterior surface of the uterus, with undistinguishable border. The uterus section appeared grey/white, solid and the texture was moderately soft. The endometrium was rather thin, with the thickness of muscularis ranging between 1.5 cm and 1.8 cm. No abnormalities were seen in the section of the uterine wall. Also size and morphology of the left adnexa showed no abnormalities (Fig. 2A). The microscopic examination of the uterus and the adnexa: It was observed that the right ovary structure was damaged, in which the tumor cells diffused and infiltrated other parts. The tumor cells, in the oval shape, seemed medium-sized or larger than the average size. There was less cytoplasm, and strong nuclear staining. The nucleus appeared circular shape, with nucleolus and nuclear division easily visible (Fig. 2B, C and D). The IHC results of the tumor tissues in the right ovary: The positive expression was detected in CD20, CD79α, PAX-5, CD 43, Bcl-6, and MUM-1. The positivity rate of P53 and Ki-67 was 20% and over 80% respectively. In contrast, negative expression was observed in CD10, Bcl-2, CD21, CD2, CD3, CD5, CD7, CD30, CD117, CD56, MP0, ER, PR, inhibin, and EBERs in situ hybridization (Fig. 3).

**Fig. 4:**
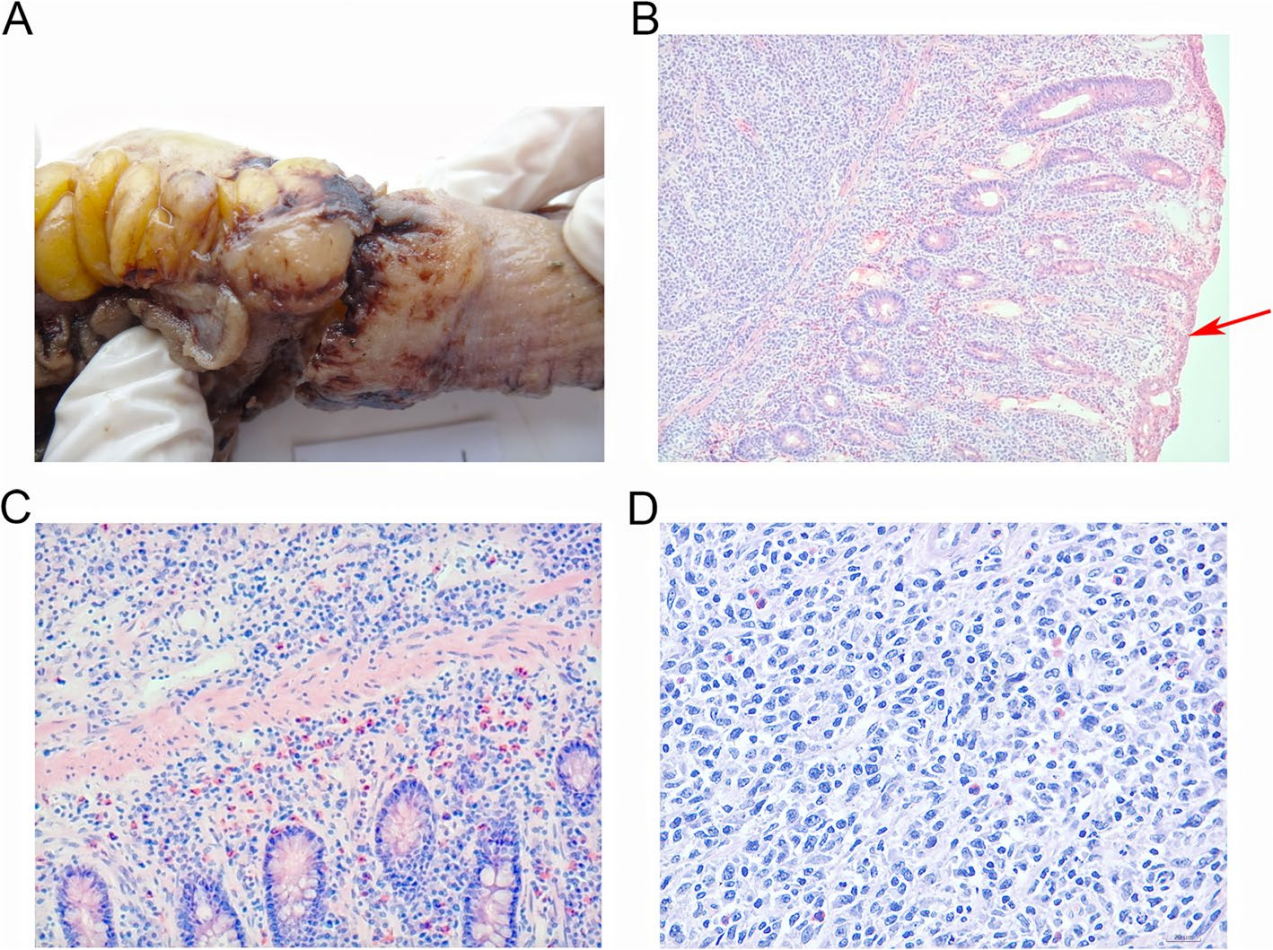
Images of excised tissues of small intestine. **A** Macroscopic image of excised tissues of small intestine. **B** The microscope revealed the intact intestinal mucosa. (H&E × 40) (see red arrows). **C** The microscope revealed the diffusion and infiltration of the tumor cells (H&E × 100). **D** The nuclear staining seemed dark, and in the staining the nuclei was noticeable and the nuclear division was obviously seen (H&E × 400). H&E, hematoxylin–eosin

**Fig. 5:**
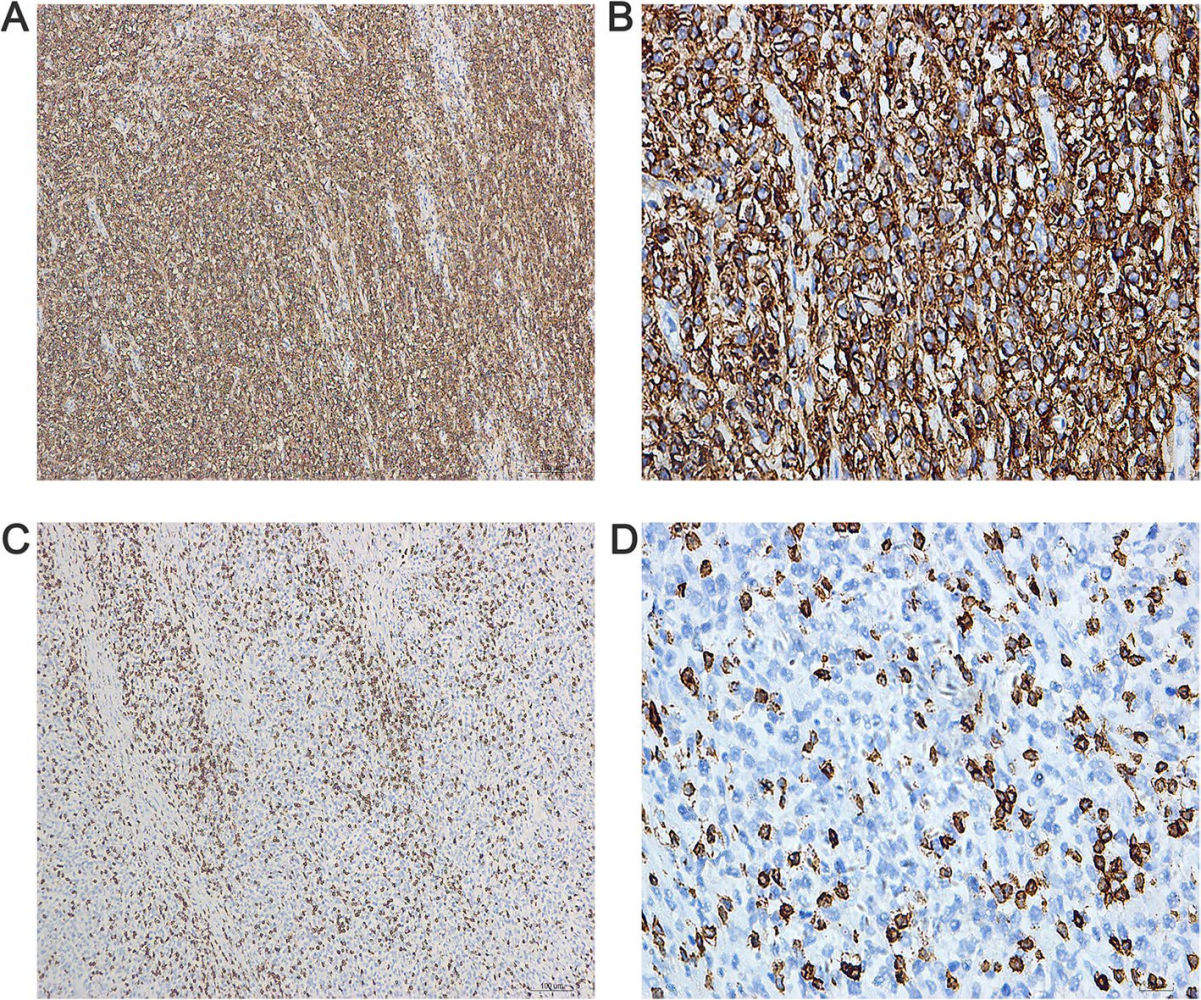
The immunohistochemical characteristics of the small intestine tissues under the microscope. In tumor tissues, cytomembrane of CD20 was positively expressed: **A** CD20 (DAB × 100). **B** CD20 (× 400). CD3 was negatively expressed: **C** CD3 (DAB × 100). **D** CD3 (× 400). DAB, Diaminobenzidine. CD3 (× 400). DAB, Diaminobenzidine

The gross examination of the small intestine: A lump, which measured approximately 1.50 cm × 1.50 cm × 1.0 cm, could be observed on the intestinal wall that was 7.0 cm away from the incisal margin at one end of the intestinal canal (approximately 15.0 cm long). The lump had somewhat indistinguishable border, spreading from the serous membrane, muscular layer of the intestinal wall to the intestinal wall. Also intestinal mucosa on which the lump was located seemed undamaged (Fig. 4A). The microscopic examination of the small intestine: The diffusion and infiltration of the tumor cells were examined on the entire small intestine wall, but the epithelial tissues of the mucosa were complete on the intestine wall (Fig. 4B) (see red arrows). The tumor cells were medium in size, and oval in shape. The nuclear staining seemed dark, and in the staining the nuclei was noticeable and the nuclear division was obviously seen (Fig. 4C and D). The IHC results of the lump on the intestine wall: The positive IHC results could be seen in CD20, CD79α, PAX-5, CD43, Bcl-6, and MUM-1; P53 had positivity rate of 15%, while Ki-67 had relatively higher positivity rate (> 80%, +). However, negative results were shown in CD10, Bcl-2, CD21, CD2, CD3, CD5, CD7, CD30, CD117, CD56, MP0, ER, PR, inhibin, and EBERs in situ hybridization (Fig. 5).

In this case, the tumor mainly invaded the entire right ovary tissues, serous membranes on the posterior surface of the uterus, and the small intestine wall. Concluding from the pathological examination results of the uterus, adnexa and small intestine, we researchers determined that the tumor in this case was aggressive malignant B-cell lymphoma. Based on the Hans classification [2] and IHC results (CD10, BCL-6, and MUM-1), the final pathological diagnosis of the case was DLBCL (germinal center B-cell-like subtype). The After the surgery, the patient underwent CT reexamination of the whole abdomen, chest and pelvic cavity. The CT scan showed a small amount of ascites and pulmonary inflammation. The patient’s pulmonary infection improved after expectant treatment. Furthermore, the laboratory test results showed no abnormalities.

## Discussion

Primary ovarian lymphoma, which is a solid tumor, has rare occurrence in female genital system. Primary ovarian lymphoma belongs to the family of extranodal lymphoma, accounting for 0.5% of NHL and 1.5% of the all ovary neoplasms [3].

At present, there is no consensus on the definition of primary ovarian lymphoma. The primary ovarian lymphoma appeared less invasive and usually had a good prognosis, whereas the secondary type progressed fast and appeared malignant. Therefore, the lesion would become a key element to differentiate between these two types of lymphoma [4]. This case applied the widely accepted diagnostic criteria proposed by Fox et al. [5] in 1988: (1) The sole clinical region was the ovary, and no lymphoma was found in other regions following the comprehensive examination (The primary lesion included the adjacent organs and lymph nodes suffering the tumor infiltration). (2) no abnormal cells were observed when examination was conducted for peripheral blood and bone marrow. (3) The secondary lymphoma of the ovary was determined if the lymphoma occurred in other organs that were distant from the ovary just several months (up to six months [6]) after the lymphoma was originally found in the ovary. (4) The patient had no history of lymphoma. (5) The malignant lymphoma was histopathologically confirmed.

Despite the low morbidity, it was still challenging to distinguish the primary ovarian lymphoma from other malignant tumors occurring in the ovary, owing to its non-specific clinic symptoms. According to the previous studies, the majority of the patients had early clinical symptoms including painless lumps in the pelvic cavity and the abdomen, abdominal pain, fever and weight loss. After reviewing the literature, we determined that this is the first case of primary ovarian lymphoma with a change in bowel habits as the first symptom. Also tumor mainly occurred in one side of the ovary, with the same size as the ovary [7]. The tumor was prone to infiltrate the adjacent tissues like the perimetrium, fallopian tube. The current diagnosis of the ovary lymphoma was mainly assisted by the biopsy; pathological diagnosis alone failed to help distinguish the ovary lymphoma from dysgerminoma, small cell carcinoma of ovary-hypercalcemic type, granulosa cell tumor of ovary and granulocytic sarcoma. But the pathological type and the cell source could be further determined by IHC staining. The primary ovarian lymphoma could be determined after the overall examination showed that no lymphomas were detected in other organs and no abnormal cells were observed in the bone marrow or peripheral blood.

DLBCL comprised the largest proportion of primary ovarian lymphoma; and according to the most frequently used Hans classification, DLBCL could be categorized into two subtypes: germinal center B-cell-like (GCB) and non-GCB, based on the immune indexes CD10, BCL-6, and MUM-1. The diseased ovary would enlarge with varying degrees, and the right part was more vulnerable than the left one with higher incidence rate. The ovary structure was damaged, and the section appeared evenly grey-white or brown-yellow, with soft texture and appearance similar to fish flesh. The histopathological examination disclosed that the tumor cells, which were monomorphic and large in size, had diffusion and infiltration, and these cells had abundant cytoplasm and obvious nuclear division.

There was no history of lymphoma in this case. The blood tests previous to the operation discovered no abnormal cells. Besides, the CT-scan of pelvic cavity illustrated that rectal cancer, together with sigmoid colon adenocarcinoma, invaded the lower part of the right-side ureter, and ureterectasis was also observed on the right. The laparotomy disclosed that the right ovary enlarged and adhered to the right fallopian tube and the pelvic wall. The tumor was seen to invade the ileum in ileocecal region, the intestine seromuscular layer in the mid-rectum, the right-side mesentery, and the lower ureter on the same side. Also laparotomy showed no observable nodules in the liver, spleen, kidneys, mesenteric root of small intestine, greater omentum, or abdominal wall. The pathological examination of the excised tissues, along with the immunohistochemistry, confirmed that the case was primary ovarian DLBCL (GCB subtype).

Considering the argument over the treatment for the primary ovarian DLBCL, the current mainstream view was to see this type of lymphoma as a local symptom of the systematic disease. Nowadays, chemotherapy was the main treatment for it, which involved multiple courses that were based on the R-CHOP regimen (Cyclophosphamide, Doxorubicin hydrochloride, Vincristine (Oncovin) and Prednisone) [8]. In addition, combined treatment was also considered effective, entailing excision, chemotherapy, and radiotherapy. The results of Vang R et al. [7] uncovered that 9 patients had a good prognosis and no recurrence when treated with chemotherapy and radiotherapy after operation. Nevertheless, Kapetanakis V et al. [9] suggested using the radiotherapy as the main treatment, cutting out the need of cytoreductive surgery. Furthermore, Weingertner AS et al. [10] pointed out that chemotheraphy combined with radiotherapy, rather than extensive excision by operation, would be more effective to guarantee a desired prognosis, and the non-operative treatment could also maintain the functions of the reproductive system. Moreover, Hu R. et al. [11] found that the positive expression of CD20 could be used as an index to indicate the combination of rituximab with CHOP, and the combined treatment would benefit the patients more.

## Conclusion

Primary ovarian DLBCL was prone to misdiagnosis owing to its rare incidence and non-specific clinical symptoms. To achieve a better diagnosis, histopathology should be cooperatively applied with IHC, the secondary lymphoma needed exclusion, and the lymphoma should be differentiated from other tumors.

In this case, we speculate that the changes in bowel habits of patient due to tumor invasion of intestinal tissue persisted for half a year. However, we have no final conclusion on whether patients with primary ovarian DLBCL are easy to invade the intestinal tract and cause changes in bowel habits. More case studies are needed to further prove this speculation.

## Data Availability

The dataset generated and analyzed within this report is available from the corresponding author upon reasonable request.

## Abbreviations

DLBCL: Diffuse large B-cell lymphoma
NHL: Non-Hodgkin lymphoma
CT: Computed tomography
H&E: Hematoxylin–eosin
IHC: Immunohistochemical
GCB: Germinal center B-cell-like
R-CHOP regimen: Rituximab, Cyclophosphamide, Doxorubicin hydrochloride, Vincristine (Oncovin) and Prednisone
